# Mandatory linking of penicillin allergy “safety alert” with beta-lactam medication history: correcting the automated inaccuracy of penicillin allergy labeling and beta-lactam antibiotic avoidance

**DOI:** 10.1017/ash.2025.10128

**Published:** 2025-11-03

**Authors:** Richard Neal Olans, David J. Witt, Ruchit Marfatia, Rita Drummond Olans

**Affiliations:** 1 IDSA Antimicrobial Stewardship Center of Excellence Consultant, Melrose Wakefield Hospital/Tufts Medicine, Melrose, MA, USA; 2 Kaiser Permanente Northern California, Mill Valley, CA, USA; 3 Antimicrobial Stewardship Co-Ordinator and Pharmacy Residency Program Director, Melrose Wakefield Hospital/Tufts Medicine, Melrose, MA, USA; 4 Associate Professor Emerita, MGH Institute of Health Professions, School of Nursing, Boston, MA, USA

Inaccurate labeling of allergies to penicillin is a medical problem of epidemic proportions, present in 10% of all U.S. patients and generally recorded as such in electronic health records (EHRs) and affecting over 30 million patients who would be predicted to tolerate penicillin or other beta-lactam antibiotics.^1,2^ The seemingly innocuous substitution of non-beta-lactam antibiotics for beta-lactams has repeatedly been associated with inferior and even fatal outcomes and promotes antibiotic resistance.^1^ Underlying causes of this epidemic are multifactorial and have been described at length.^2^ They began following initial descriptions of anaphylaxis in the late 1940’s. These reports were frightening both to physicians and to the public, resulting in cautionary warnings to avoid penicillin at all costs. Family legends were fashioned to shun penicillin forever.^3^

The inaccurate penicillin allergy label problem worsened with expansion of the EHR. As part of the meaningful use provisions of the Health Information Technology for Economic and Clinical Health Act of 2009,^4^ developers of EHRs were required to protect patient safety by reconciling patients’ past and current medications and allergies. Programmers developed safety alerts including “Allergic to Penicillin” pop-ups present in all EHR systems. Unfortunately, such alerts were configured to be triggered by any previous listing of penicillin allergy without specific description or diagnostic validation. These alerts were frequently based on unverified patient histories or on non-allergic adverse events such as nausea. These are typically not updated or corrected even when a patient has contradictory evidence in the same EHR documenting their having received penicillin without adverse reaction.^5^

Even the name “Penicillin Allergy Safety Alert” belies the purposes of its function. Analysis of the name reveals a four-part misnomer as follows:

*Penicillin*: The alert is triggered not only when penicillin is prescribed but also pops up when other beta-lactam antibiotics are prescribed. Cephalosporins were originally thought to be 8%–10% cross-reactive with penicillin. With postmarketing surveillance and improved purification during production and appreciation of specific cephalosporin side chain structures, cross-reactive allergy was found to be only 1% in persons with an unverified history of penicillin allergy and 2% in persons with confirmed positive penicillin skin tests.^6^ Despite this current data, the net effect of the current safety alert is to warn prescribers away from ordering all beta-lactam antibiotics. This migration represents more than a mere electronic inconvenience. Elimination of all beta-lactam antibiotics removes over fifty of the most useful antibacterial agents in the therapeutic armamentarium, including treatments of choice for certain pathogens and for several clinical syndromes. Alternative or second choice agents do not represent innocuous swaps and often lack equivalent therapeutic efficacy.^1^

*Allergy:* Alerts identify allergy even when the supposed event is a non-allergic condition, such as nausea, fatigue, or even unknown. Even when allergy has been disproven, the allergy alert remains uncorrected.^5^

*Safety:* In reviews of over a dozen published series with over 2,000 total patients labeled as penicillin allergic but nonetheless treated with beta-lactam antibiotics after risk-stratified screening, no fatalities or serious complications occurred.^7,8^ On the other hand, avoidance of beta-lactam antibiotics has repeatedly been associated with inferior and even fatal outcomes. These include the treatment of staphylococcal^9^ and gram negative bacteremia,^10^ less effective surgical prophylaxis leading to postoperative infections,^11^ treatment failures in immunocompromised patients,^12^ increased lengths of stay and readmissions, and selection of multidrug-resistant pathogens.^13^

*Alerts:* By failing to reconcile discordant prior safe receipt of beta-lactam antibiotics, the pop-up notifies clinicians with allergy misinformation while failing to alert clinicians of relevant beta-lactam tolerance. The alert thus misleads more than it alerts. Yet its abundant repetition has given the term “label” a false veneer of diagnostic reality. Its sole defining characteristic is its inaccuracy. When challenged, the penicillin allergy label is shown to be inaccurate 90% of the time.^1,7^

In response to the awareness of these deficiencies, numerous institutions have attempted to work around this misdiagnosis.^2,7^ Removing the label at one site, however, does not update or permanently amend the penicillin allergy safety alert from subsequent visits, transferred sites, or on different EHR platforms.^5^ This process, called delabeling, often proves unsustainable in practice, with 36%–49% of delabeled inpatients continuing to be relabeled within one year.^14^ Numerous articles have been published about inaccurate EHR penicillin allergy labels. Most of these papers focus on correcting this by removing the “Allergy to Penicillin” label. It sequesters any evidence of mislabeling in the allergy module rather than addressing the persistent inaccurately relabeled beta-lactam alert’s influence on human, professional, and societal behaviors. Even in centers with established delabeling programs, relabeling outpaces delabeling,^13^ and prescribing misperceptions with harmful consequences persist.

Why is the acknowledged erroneous safety alert more impactful than factual medication records? Factually accurate discordant prior tolerance of beta-lactams are not displayed as are the embedded inaccurate “Allergy/Contraindication: Penicillin” alerts. The pop-up remains a powerful dogmatic behavioral nudge despite extensive publications demonstrating its safety/tolerance after test challenge over 90% of the time.^7^ The ubiquity of the EHR, with the immediacy of its alert with every beta-lactam antibiotic order, are powerful deterrents to the careful preprescribing risk/benefit analysis supposedly inherent in every prescribing event.^15^ The pop-up supersedes the dozens of published articles that uniformly decry the allergy inaccuracy and powerfully misdirects human prescribing behavior. It is difficult to dislodge long-held beliefs or to change confirmational biases.^16^ Antibiotic prescribers and monitors (nurses) have been indoctrinated to believe these false alarms; they must be re-educated how to clinically react differently.

The electronic health record was hoped to facilitate improvement of care. Unfortunately, the penicillin allergy alert usurps critical thinking. The current alert design functions not as Augmented Intelligence but as a persistent Automated Inaccuracy. Rather than a Clinical Decision Support System, it performs as a Computer Driven Stigmatizing System. Although the EHR did not perpetrate the original penicillin allergy mislabel, its current configuration contributes to this mis-diagnosis epidemic.^2^ Public misperceptions, patient self-relabeling via patient portals, and the programmatic re-entry of the EHR allergy alert are major contributors driving the outpacing of delabeling by relabeling.^3^

The alert could readily be corrected to guide appropriate practice. Straightforward interventions could help curb this epidemic of mislabeling.

*First:* EHR allergy alerts for beta-lactam antibiotics must be mandated to reconcile allergy with medication histories. Relevant prior allergy and medication data are functionalities already present in existing EHRs, but both must be simultaneously displayed as part of a meaningful two-part alert. Specific information regarding tolerance or penicillin skin testing should be identified by a prescriber friendly, single-click link to the precise details of the beta-lactam tolerant event, permitting safer, more accurately informed beta-lactam antibiotic prescribing. This could also be augmented by an automated presentation of a risk-assessment tool as well.^8^

*Second:* Remove the routine automatic allergy alert for cephalosporins for every penicillin allergy label. Serious allergic cross reactions for cephalosporins among those with a label of penicillin allergy are extremely uncommon, particularly with cefazolin with its unique side chain.^1,6^

*Third:* Create a diagnostic code for patients who have tolerated beta-lactam antibiotics despite prior report of penicillin allergy. A clear and specific diagnostic code/digital marker would be useful for timely identification and documentation of mislabeled individuals who had been proven to be beta-lactam antibiotic tolerant. This could guide subsequent optimal antibiotic management. Current codes for “Mis-diagnosis” may be unpalatable to many prescribers. We suggest a diagnostic acronym of Beta-Lactam antibiotic Avoidance: Tested Allergy Now Tolerant (BLATANT) syndrome. This accurately describes the scope of the medical error and affirms the safety of prescribing a beta-lactam. Such a CPT code could be used by the increasing numbers of existing delabeling sites^17^ and antimicrobial stewardship programs to durably document safe tolerance events across disparate EHR platforms. This could provide a tool to restore safer, more evidence-based risk/benefit antibiotic prescribing practices.^2^

Delabeling partnerships between existing antimicrobial stewardship programs and clinicians already improve patient outcomes and prevent harmful consequences. These include anesthesiology, surgery, emergency medicine, hospital medicine, obstetrics and pediatrics departments.^1,7^ But despite this, sustained delabeling is not generally preserved beyond the initial/parent institution.^2,14^ Documentation with a tolerance diagnostic code (BLATANT) following completion of safe beta-lactam treatment could provide both short- and long-term reassurance for patients and prescribers.

We are not dealing with an epidemic of anaphylaxis, rather a pervasive endemic of penicillin allergy misinformation. Every prescribing event requires thoughtful risk/benefit analysis. Making such decisions misdirected by a flawed algorithm distorts the entire therapeutic decision-making process.^2^ Without accurate information, all antibiotic prescribers, physicians, pharmacists, and nurses are denied accurate evidence-based information on which to make safe patient treatment decisions.

Scientific evidence supporting the need for correction of inaccurate penicillin allergy is abundant and widely accepted.^1,7^ Lacking are immediate implementation strategies to effect necessary change. These require cooperation from the EHR industry to accomplish a more accurate allergy alert. This is not an issue of proprietary product protection^18^ but, at least in patients with documented discordant beta-lactam allergy alerts but tolerant medication data, it is an issue of demonstrably harmful algorithm design. Our recommendations should not be misconstrued as a demand to abolish all allergy safety alerts but rather as a call to action to correct the current flawed design of penicillin allergy alerts. Such corrective change offers EHR designers the opportunity to be seen as part of the solution rather than as part of the problem.^2^ The ever-present EHR format that currently misleads prescribers into inappropriate beta-lactam antibiotic avoidance could be transformed into a reminder enlisting nurses, pharmacists, and other frontline healthcare workers into useful, fact-based patient educators. The incentive for regulation is not only quality-driven but also offers savings of millions of dollars currently wasted annually.^2,19^

Restructuring of the inaccurate penicillin/beta-lactam safety alert to a more evidence-based configuration is long overdue. Clearly, antibiotic allergy education and pharmacology education are necessary in reversing this misinformation epidemic. To date, however, neither simple label removal, robust EHR allergy sections, patient wallet cards, nor detailed patient histories have proven broadly and sustainably corrective. We postulate that required EHR linking of penicillin allergy labels simultaneously published with past beta-lactam exposure can begin to improve the entire allergy review process in a manner that can successfully redirect the allergy mislabeling culture.

A recent review acknowledges the “need to identify optimal interventions and implementation approaches” to improve the quality of antimicrobial stewardship performance.^20^ Our proposed interventions could amplify efforts of current delabeling programs to more accurately approach the clinical practice and management of penicillin allergy. This can interrupt the prevalent progression from public and professional penicillin misperceptions to misleading safety alerts and ultimately to inferior antibiotic management. It can harness the ubiquity of the EHR and the repetitive immediacy of the alert prompt to change the current beta-lactam avoidance habit and to restore a more rational, evidence-based antibiotic prescribing behavior. Such a process, paired with educational and antibiotic stewardship efforts, can help achieve optimal antimicrobial stewardship outcomes and educational goals while improving both individual patient safety and public health outcomes.
